# Rapid Assessment of Binding Affinity of SARS-COV-2 Spike Protein to the Human Angiotensin-Converting Enzyme 2 Receptor and to Neutralizing Biomolecules Based on Computer Simulations

**DOI:** 10.3389/fimmu.2021.730099

**Published:** 2021-11-11

**Authors:** Damiano Buratto, Abhishek Saxena, Qun Ji, Guang Yang, Sergio Pantano, Francesco Zonta

**Affiliations:** ^1^ Shanghai Institute for Advanced Immunochemical Studies, ShanghaiTech University, Shanghai, China; ^2^ Institut Pasteur de Montevideo, Montevideo, Uruguay

**Keywords:** COVID-19, SARS-CoV-2, Spike-RBD, human ACE2, binding affinity, neutralizing antibodies, protein-protein interaction, molecular dynamics

## Abstract

SARS-CoV-2 infects humans and causes Coronavirus disease 2019 (COVID-19). The S1 domain of the spike glycoprotein of SARS-CoV-2 binds to human angiotensin-converting enzyme 2 (hACE2) *via* its receptor-binding domain, while the S2 domain facilitates fusion between the virus and the host cell membrane for entry. The spike glycoprotein of circulating SARS-CoV-2 genomes is a mutation hotspot. Some mutations may affect the binding affinity for hACE2, while others may modulate S-glycoprotein expression, or they could result in a virus that can escape from antibodies generated by infection with the original variant or by vaccination. Since a large number of variants are emerging, it is of vital importance to be able to rapidly assess their characteristics: while changes of binding affinity alone do not always cause direct advantages for the virus, they still can provide important insights on where the evolutionary pressure is directed. Here, we propose a simple and cost-effective computational protocol based on Molecular Dynamics simulations to rapidly screen the ability of mutated spike protein to bind to the hACE2 receptor and selected neutralizing biomolecules. Our results show that it is possible to achieve rapid and reliable predictions of binding affinities. A similar approach can be used to perform preliminary screenings of the potential effects of S-RBD mutations, helping to prioritize the more time-consuming and expensive experimental work.

## Introduction

Severe acute respiratory syndrome coronavirus-2 (SARS-CoV-2) causes pneumonia/severe respiratory infection in humans called Coronavirus disease 2019 (COVID-19). The first cases of COVID-19 were reported in December 2019 from Wuhan, China (1). At the moment of writing this manuscript, SARS-CoV-2 infection is reported in ~180 million people resulting in ~5 million deaths (2). SARS-CoV-2 is an enveloped virus belonging to a diverse subgenus sarbecovirus within the Betacoronaviruses, a lineage of viruses that use bats as reservoirs and can be transmitted into other mammals (3–8). SARS-CoV-2 is genetically distinct from severe acute respiratory syndrome coronavirus (SARS-CoV) and Middle East respiratory syndrome coronavirus (MERS-CoV). Its closest known relative is Bat CoV RaTG13 (4–6), with 96.3% of gene identity. The single-stranded RNA genome of SARS-CoV-2 encodes Spike (S), Envelope (E), Membrane (M), and Nucleocapsid (N) structural proteins (5). The Spike (S) glycoprotein comprising S1 and S2 subdomains interacts with human angiotensin-converting enzyme 2 (hACE2) present primarily on pneumocytes/lung immune cells for attachment (*via* S1 c-terminal receptor-binding domain S-RBD), fusion, and virus entry into the host cell (*via* S2) (9–12). S-RBD also has a role in cross-species transmission and evolution of SARS-CoV-2 (8, 12–15). It is also the major immune determinant of a human neutralizing immune response upon natural infection and vaccination (16–19). Although S-RBD plays a critical role in viral infectivity and transmission, it is highly variable among sarbecoviruses and possibly a hotspot of complex selective pressure which shapes SARS-CoV-2 evolution (8, 20–22). Recombination events in the genome contribute to CoVs evolution, and recombination breakpoints are evident in the SARS-CoV-2 genome at the beginning and end of the S-RBD (6, 9, 23). Mutations within the S-RBD can increase affinity for ACE2, transmissibility, and mediate immune escape (8, 24–28).

Computer simulations have been widely used to provide important insight into the role of mutations within the S-RBD. Molecular dynamics (MD) simulations-based analyses show some of the earliest known S-RBD mutations (F342L, N352D/D364Y, V367F, W436R, and V483A) can increase binding affinities and favor hACE2 interaction (29–31). Another study on the B.1.135 (K417N/E484K/N501Y) variant suggests that while N501Y alone could improve the binding affinity, the other two mutations reduced it, possibly causing a non-net change in binding properties (26). In agreement with this hypothesis, experimental mutational scanning suggest that N501Y/N501T slightly increase hACE2 binding while K417N/K417T enhance S-RBD expression, and E484K did not cause any significant phenotypic change (8).

In this paper, we report a computational protocol to rapidly assess the binding affinity of the S-RBD to the hACE2 receptor. We applied our method on some early reported mutations (G476S, V483A, H519Q, and H520), the triple mutant B.1.135 K417N/E484K/N501Y, first isolated in South Africa – beta variant, and triple mutant P.1 K417T/E484K/N501Y, first isolated in Brazil – gamma variant, investigating their effect at the molecular level. Our results suggest that none of the mutations causes an essential increase of the *K_D_/IC_50_* properties of the Spike protein. Still, we observe a significant, albeit small, decrease of binding affinity for the two triple mutants. However, we observe a more marked reduction of the binding of spike protein to an artificial neutralizing nanobody caused by E484K, a mutation found in several variants of concern and which has been reported to produce a virus able to escape neutralizing antibodies (32–35).

## Material and Methods

### Molecular Modeling and Dynamics

The model of the reference type (RT) variant Covid-19 RBD in complex with hACE2 receptor was derived by the X-RAY crystal structure PDBID 6LZG (36). All the mutants of the Covid-19 S-RBD were created starting from this model using CHIMERA (37). Each model was solvated with TIP3P water, containing Cl- and K+ ions at a concentration of ∼0.15 M to mimic the physiological ionic strength. After solvation, the total number of atoms for each system was around 1.7 × 10^5^.

MD simulations were carried on using the Gromacs 2018 package (38) and the Amber14SB force field (39), following simulation protocols similar to those we used in our previous works (40–43). Specifically, after energy minimization, we performed 200 ps of Simulated Annealing to allow side chains to equilibrate after each mutation is introduced. We then performed two short simulations lasting 100 ps, first in the NVT and then in the NPT ensembles, both with positional restraints (being the position restraint constant $k_{pr}=1000\frac{KJ}{mol\cdot nm^{2}}$ ) on the heavy atoms of the protein. Finally, we performed equilibrium MD simulation under periodic boundary conditions at constant pressure for 50 ns. Analyses were performed only on the last 25 ns after equilibration, as explained in the Results section. Temperature T and pressure P were kept constant during the equilibrium MD simulation, at 300 K and 1 atm, respectively, using the Berendsen thermostat and barostat (44). Fast smooth Particle–Mesh Ewald summation (45) was used for long-range electrostatic interactions, with a cut-off of 1.0 nm for the direct interactions. Each simulation was performed in five identical replicas: while classical MD simulations are in principle deterministic, parallel computing algorithms currently implemented in MD software can produce different trajectories. Nevertheless, experimental structures represent a thermodynamic average. Hence, replicating the simulations allows to check the results consistency and reduce the risk of being trapped by entropic barriers, thus improving the sampling of the configuration space available.

### Binding Free Energy Computations

To produce fast and reliable predictions of the binding free energy, we use the PRODIGY web server (46, 47), which has been designed for this purpose. The results are then compared with those obtained with MM-PBSA (an acronym for Molecular Mechanics – Poisson Boltzmann and Surface Area continuum solvation approximation method), a more standard methodology, widely used in the field (48, 49). Binding free energies are calculated as ensemble averages over the configuration space explored by the five different replicas. To speed up the calculation, while maintaining a meaningful set of configurations for the energy calculations, we clustered the configuration space sampled by the various MD trajectories after equilibration (i.e., the last 25 ns each of the five replicas) according to their root mean square deviation (RMSD), and calculate the binding energy using one representative for 60 bigger clusters, being the clustering distance 1.2 Å. The final result is then obtained as the average of the free energy computed for each of these configurations. The results obtained by the two methods show correlation (R=0.82, **Supplementary Figure 1**). However, the PRODIGY webserver is considerably faster than the MM-PBSA calculations (~50 times faster than our local MD-dedicated GPU cluster - this figure can be much higher if parallel computational resources are not available). Furthermore, it requires a much easier set-up so that the calculation can be performed by less experienced investigators.

From the binding free energy difference, it is possible to estimate the change in binding affinity using thermodynamics theory, according to the expression (50): $RT\Delta \ln\left(K_{D}\right)\;=\;\Delta \Delta G_{Exp}$ . The results computed with the PRODIGY webserver show correlation with experimental data (**Supplementary Figure 2**).

### Recombinant Production of SARS-CoV-2 S-RBD Mutants and hACE2

Reference SARS-CoV-2 S-RBD (nt 22,517 – 23,185; MN908947) and hACE2-ECD (aa19 – aa617; Uniprot Q9BYF1) coding gene fragments were obtained by chemical synthesis. S-RBD reference gene with C-terminus hexahistidine tag was cloned in *5’NotI/3’BamHI* restriction sites of pSCSTa plasmid under the control of CMV promoter and used to generate mutant constructs (G476S, V483A, H519Q, and A520S) by oligonucleotide-mediated PCR mutagenesis. All S-RBD proteins were transiently produced in FreeStyle 293f cells (Invitrogen). Culture supernatant containing protein was bound to Ni-NTA resin (Yeason Biotech), eluted with 500 mM imidazole in 20 mM HEPES/500 mM NaCl, buffer exchanged to 1x PBS and further cleaned by size exclusion chromatography using Superdex 200 10/300 column (GE Healthcare) on AKTA Avant150 FPLC system. The Human ACE2 gene was cloned into unique *SfiI* restriction sites in pFUSE-mIgG2A-Fc2 plasmid (Invivogen) under the control of a hEF1-HTLV-1 promoter and produced like S-RBD proteins. Culture supernatant containing hACE2-ECD-mFc was bound to Mabselect resin (GE Healthcare), eluted by Pierce IgG elution buffer (Thermo Scientific), and buffer exchanged to 1x PBS. All recombinant proteins were resolved on 4 – 12% gradient SDS-PAGE to ascertain purity and correct size.

### Experimental Determination of the Binding Affinities Between S-RBD Variants and ACE2 Receptor

Maxisorp ELISA wells were coated with 200 nM S-RBD reference/mutant protein overnight at 4°C, blocked with 2% (w/v) skimmed milk at room temperature for 1 hour (h). To determine *EC_50_* values, hACE2-ECD dilutions (two-fold; 250 nM – 0.015 nM) were added to the designated wells and incubated at room temperature for 1 h. To determine *IC_50_* values, hACE2-ECD at predetermined *EC_50_* concentration (for respective S-RBD reference/mutant) was mixed with the cognate S-RBD protein (two-fold dilution; 1000 – 0.487 nM) and incubated at room temperature for 1 h before being added to the designated S-RBD reference/mutant coated and blocked wells. The binding was detected with 1:1000 diluted anti-mouse IgG (Fc specific) PO labeled secondary antibody (Cell Signalling Technologies).

The kinetics of S-RBD – hACE2-ECD interaction was analyzed by biolayer interferometry (BLI) using the Octet Red96 system (PALL ForteBio). HIS1K dip and read optical sensors (PALL ForteBio) were used to detect non-specific binding with the highest concentration of hACE2-ECD used in the assay, and passed sensors were subsequently loaded with 1000 nM S-RBD reference/mutant protein to reach a loading threshold of ~0.5 nm. Human ACE2-ECD dilutions (two-fold; 2500 – 312.5 nM) were used as an analyte to measure *KD*. Reference sensors with no load and reference well with only 1x kinetics buffer were used as controls.

### Statistical Analysis

Statistical analysis of ELISA data was done using Prism software version 8.00 (GraphPad). *EC_50_* and *IC_50_* values were determined by nonlinear regression analysis, by fitting log (agonist concentration) vs. response and log (inhibitor concentration) *vs*. normalized response, respectively.

The *IC_50_* binding curves (column means) were analyzed by two-way ANOVA with Dunnett’s multiple comparison tests. The biolayer interferometry (BLI) binding curves were generated and *K_D_* were determined by fitting the curves globally and analyzed by the 1:1 model using Pall Forte Bio Octet Data Analysis Software version 10.0. The *K_D_* values of three replicates so obtained were compared by two-way ANOVA with Tukey’s multiple comparison tests using Prism software version 8.00 (GraphPad).

Binding free energies were computed from the representative configuration of the 60 more populated cluster. Values on **Tables 1**, **2** are presented as averages and standard errors of the mean. The errors for the variation of binding affinity were computed with the error propagation formula, p-values were obtained using the Student t-test. Box plots were draw using Python and the Seaborn package.

**Table 1:** Table of computed ΔG and ΔΔG (ΔG_variant_ – ΔG_RT_).

	ΔG kcal/mol	σ_ΔG_ kcal/mol	ΔΔG kcal/mol	p-value
Reference Type	-12.02	0.06	–	–
G476S	-11.40	0.08	0.61	7.8*10^-8^
V483A	-12.15	0.08	-0.13	0.21
H519Q	-12.18	0.08	-0.17	0.11
A520S	-11.88	0.08	0.13	0.19
N501Y E484K K417N	-11.11	0.07	0.90	3.7*10^-16^
K417N	-11.97	0.08	0.05	0.62
N501Y E484K K417T	-11.03	0.09	0.98	3.3*10^-15^
K417T	-11.84	0.07	0.17	0.08
N501Y	-11.63	0.08	0.39	2.5*10^-4^
E484K	-12.10	0.08	-0.09	0.41

The table reports averages, standard error of the mean and p-values of the difference between the binding affinities of S-RBD and hACE2 receptor. The relative binding affinity **ΔΔG** use the RT as reference.

**Table 2:** Table of computed ΔG and ΔΔG (ΔG_gamma variant_ – ΔG_RT_) to neutralizing proteins.

	ΔG kcal/mol	σ_ΔG_ kcal/mol	ΔΔG kcal/mol	p-value
RT - nanoBody (7JVB)	-10.36	0.09	–	–
Gamma - nanoBody (7JVB)	-9.13	0.06	1.23	3.0*10^-17^
RT - miniprotein (7JZM)	-9.19	0.05	–	–
Gamma - miniprotein (7JZM)	-9.06	0.05	0.13	0.06
RT - miniprotein (7JZU)	-9.92	0.06	–	–
Gamma - miniprotein (7JZU)	-9.70	0.04	0.23	1.1*10^-3^

The table reports averages, error of the mean and p-values of the difference between the binding affinities of gamma variant S-RBD and different neutralizing proteins. The relative binding affinity **ΔΔG** use the RT as reference.

## Results

### Sampling of the Interaction Between the S-RBD and the hACE2 Receptor

A reliable computation of the binding free energy between two proteins should take into account the possibility of their dynamical rearrangement and extensive sampling (50). X-ray structures can be considered as faithful representations of the energy minima, but cannot take into account a very important contribution to their binding affinity, i.e., the temperature effects on the two interacting proteins. Furthermore, when introducing a mutation in a structural model, it is likely that the local structure will not be well equilibrated. For both these reasons, we performed MD simulations of each possible pairs of spike-hACE2 receptor proteins. Each simulation was repeated in five different replicas to further improve the configurational sampling and reducing the probability of being trapped in local minima (see Methods section).

Analysis of the root mean square deviation (RMSD - **Supplementary Figure 3**) of the various trajectories shows that the S-RBD finds its equilibrium position on average after 25 ns. For this reason, we decided to carry on the following analysis on the second half of each trajectory.

**Figure 1:**
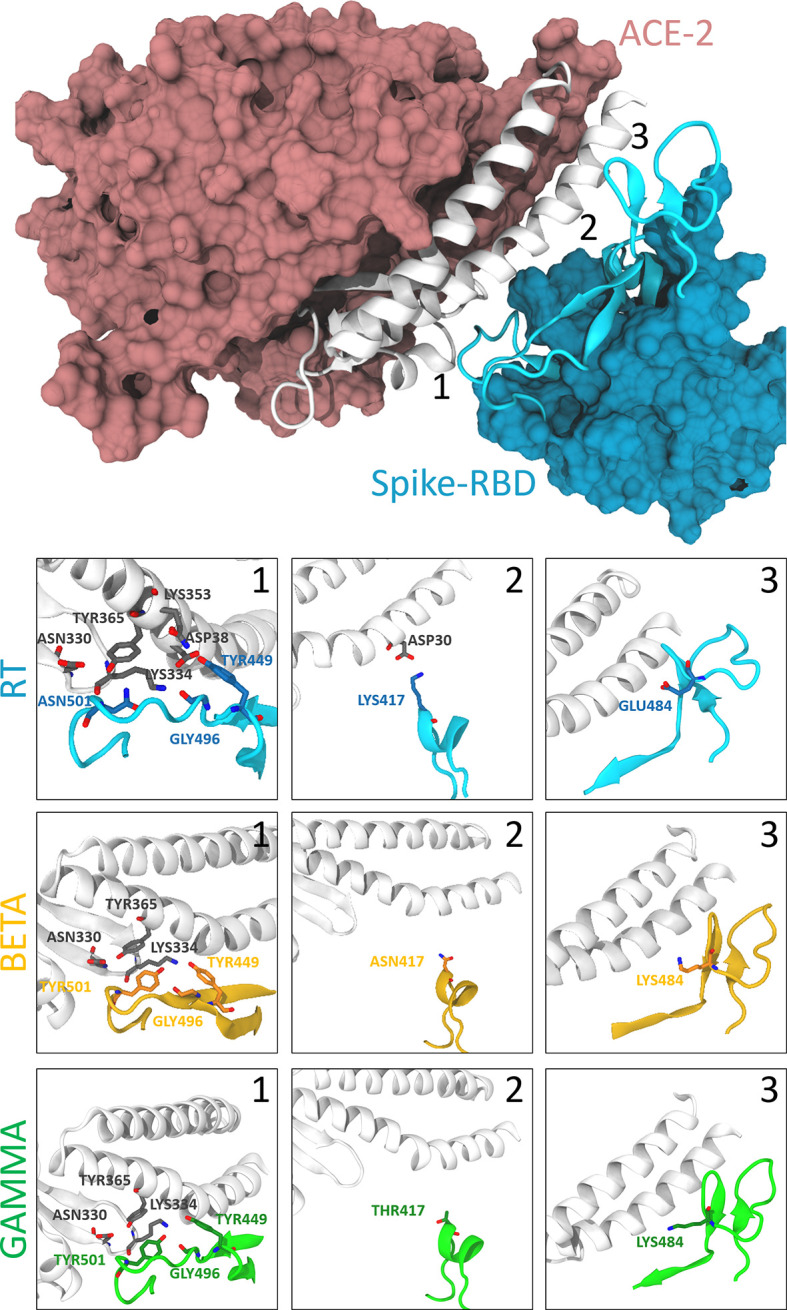
Interactions between the spike protein and the hACE2 receptor. The top panel shows the binding between the S-RBD region of the RT and the hACE2 receptor. The interaction interface is shown in cartoon representation (white for hACE2 and cyan for S-RBD), while the rest of the protein is represented according to its surface (pink for hACE2 and light blue for S-RBD). The bottom panels show the differences between RT (cyan), beta (orange), and gamma (green) variants in correspondence with the position of the three S-RBD mutations (labeled with numbers 1-3 in the top panel). Relevant residues are shown with their side-chain representation in these panels. One can notice how residue Asn501 is at the center of a rich pattern of interactions, which is altered after its mutation to Tyr. On the other hand, Lys417 of the S-RBD interacts only with the Asp30 of the hACE2, and this interaction is broken after its modification to a non-basic amino acid. Finally, Glu484 does not show any critical interactions, and this does not change with the mutants.

From the dynamic point of view, all the different variants behave similarly. Analysis of the contact maps between the two proteins reveals that in the RT variant, the interaction is mainly mediated by residues Lys417, Tyr449, Leu455, Phe456, Ala475, Phe 486, Asn487, Tyr489, Gln493, Gly496, Gln498, Thr500, Asn501, Gly502 and Tyr505 (interaction probability higher than 90% along the trajectory, see **Figure 1**, **Supplementary Figures 4**, **5**). No significant difference is observed between the RT and the single point mutations G476S, V483A, H519Q, A520S. It is worth noticing that only the first two mutants are in proximity of the binding region, while the other two are far from it, and we do not expect to see any effect on the binding to the hACE2 receptor caused by them.

When looking at the two triple mutants, we can observe that mutations of Lys417 and Asp501 are slightly more impactful in affecting the interaction between the spike protein and the hACE2 receptor. Lys417 forms a salt bridge with Asp30 of hACE2, which is abolished upon lysine mutation to asparagine or threonine. The change from asparagine to tyrosine in position 501 forces a different arrangement of the spike protein residues Tyr499 and Gly496, affecting their interactions with Asp38, Gln32, and Lys353 of the hACE2 (**Figure 1** and **Supplementary Figure 5**). Since Glu48 does not interact with the hACE2 receptor in the RT, its mutation to lysine does not produce critical differences in the contact map.

**Figure 2:**
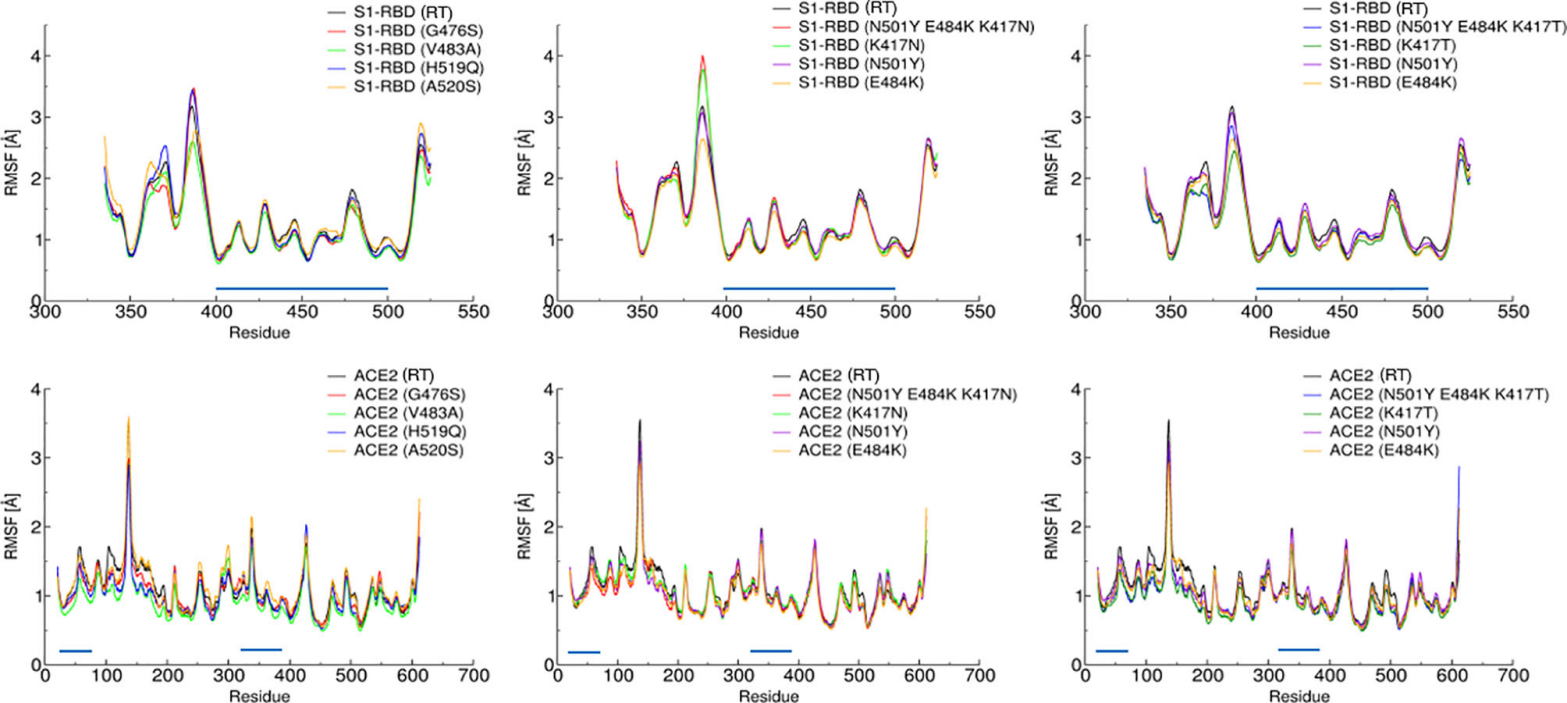
Root Mean Square Fluctuation of the S-RBD variants (top panels) and hACE2 receptor (bottom panels) vs. residue index. The graphs are divided into three different panels for better readability. Amino acids belonging to the contact region are indicated by a blue line parallel to the x-axis. A direct comparison between the various trajectories reveals that the dynamic behavior of the complex S-RBD/hACE2 is not significantly affected by these mutations.

In agreement with these observations, root mean square fluctuations (RMSF) show no evident changes in the dynamical behavior of the complex, especially in the contact region (**Figure 2**).

### Rapid Evaluation of Binding Free Energy Between the S-RBD and the hACE2 Receptor

In principle, binding free energy estimates can be computed from each of the configurations obtained from MD simulations. However, the computational cost for repeating the calculation on all of them would be extremely high, especially if we use standard methodology like MM-PBSA. Moreover, MD trajectories may be highly correlated on the short time scale. Hence, to ensure we are considering a wide variety of configurations, we clustered them using a 0.12 nm RMSD cutoff, and we computed the binding free energy only for one representative in each of the 60 bigger clusters. The final estimate is obtained as the average of the 60 representative configuration.

To further reduce the time of computation of the binding free energy, we decided to use the PRODIGY web server (46), which produces results comparable with MM-PBSA calculations, being at the same time much less computationally expensive.

**Figure 3:**
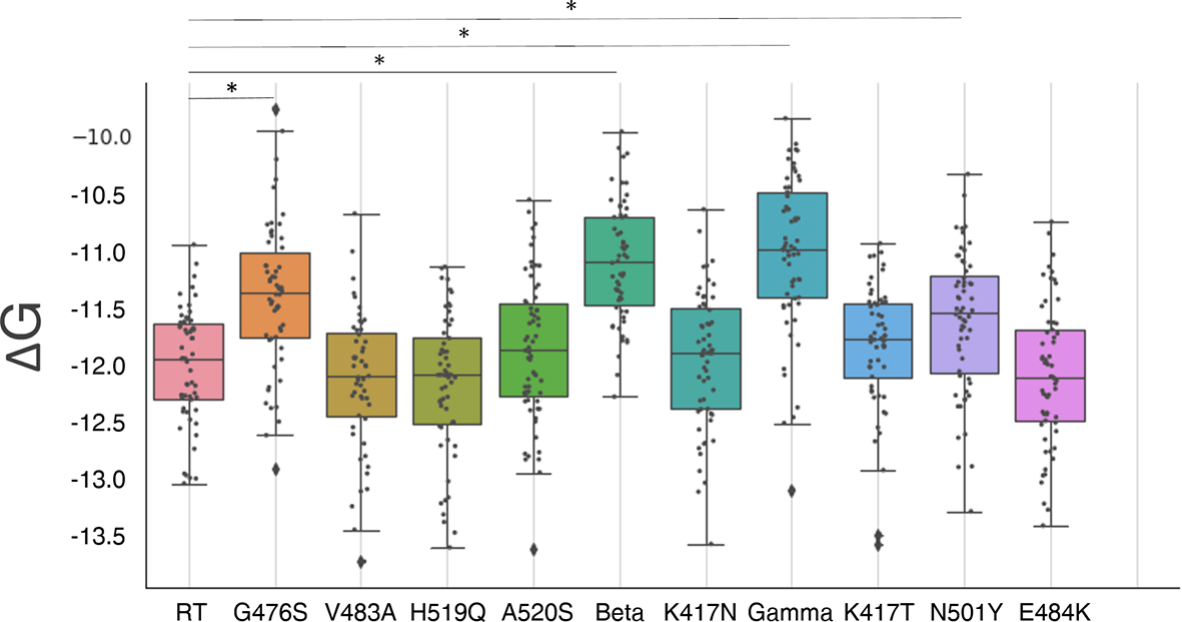
Box plots of the distribution of S-RBD and hACE2 receptor binding free energy. A boxplot is constructed of two parts, a box and a set of whiskers. The box is drawn from the first quartile (*Q*_1_, the median of the lower half of the dataset) to the third quartile (*Q*_3_, the median of the upper half of the dataset) with a horizontal line drawn in the middle to denote the median. The whiskers are drawn from the upper/lower quartile to the largest/lowest data point excluding any outliers. The outliers are shown with black diamonds. Statistically different distributions are indicated with a (*) symbol. G476 mutant, alpha (N501Y), beta, and gamma variants show a worse binding affinity. However, the differences in absolute value are small.

Results are reported in **Figure 3** and **Table 1** (free energy differences with the RT).

None of the mutants show a significantly improved binding affinity, while mutants G476S, N501Y (alpha variant), and the two triple mutants (beta and gamma variants) show a significantly decreased binding affinity (P<<0.05) with a binding energy difference of 0.6 ± 0.8 kcal/mol, 0.4 ± 0.8 kcal/mol, 0.9 ± 0.7 kcal/mol, and 1.0 ± 0.8 kcal/mol respectively. These results are compatible with similar studies on the triple mutants (26, 51). All the other mutants show no significant differences (P>0.08). However, in all the cases the changes are small (less than 1kcal/mol), and the binding affinity changes are predicted to be within a 5-fold range.

### Mutation E484K Reduces the Binding Affinity of the S Protein to a Potent Neutralizing Nanobody

**Figure 4:**
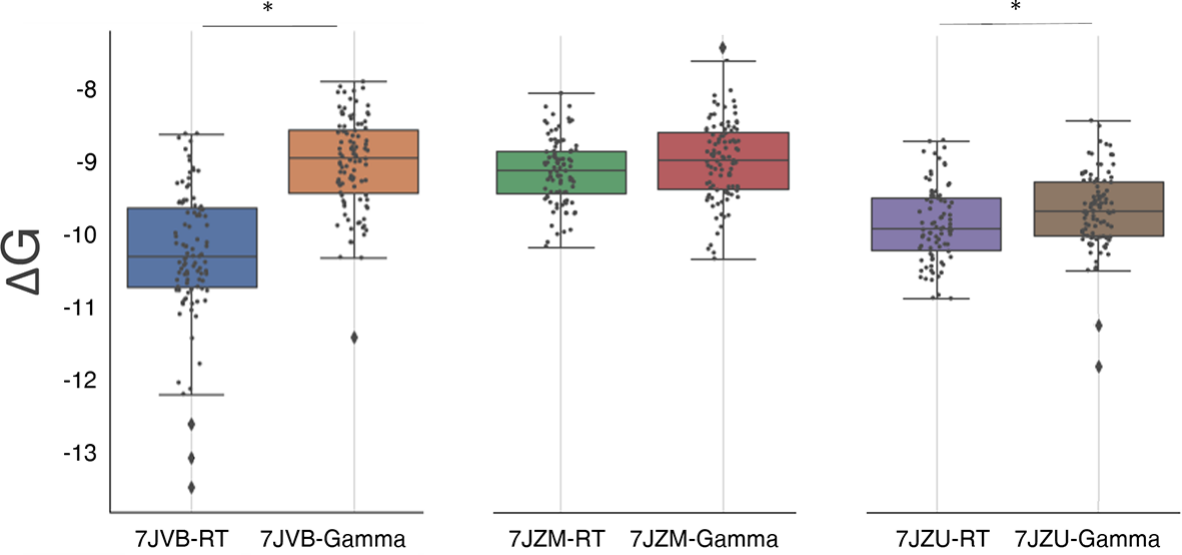
Box plots of the distribution of S-RBD and neutralizing molecules binding free energy (see **Figure 3** for the box plot description). The three plots compare the binding free energy of the RT and the gamma variant to three neutralizing molecules (described in the text). Statistically different distributions are indicated with a (*) symbol.

To test whether spike mutations can result in a virus that is able to escape immune response, we explored the effect of the mutation present in the gamma variant on the binding affinity of the highly specific nanobody Nb20 (PDB ID 7JVB) reported by Xiang et al. (52). We also tested different kinds of neutralizing molecules, i.e., the highly specific miniproteins LCB1/LCB3 (PDB ID 7JZU and 7JZM respectively) designed by Cao et al. (53), using the same method described in the previous section. Our calculations revealed that the affinity of the nanobody to the gamma variant is significantly decreased when compared to the RT, with a difference in the binding energy of 1.2 ± 0.1 kcal/mol, which translates into approximately a 7.5-fold decrease of binding affinity. This may be in agreement with the significant reduction in binding affinity reported for this nanobody against the E484K mutation (54). The interaction of the miniproteins LCB1 and LCB3 with the gamma variant shows a small increase of 0.13 ± 0.06 kcal/mol (P=0.06) and 0.23 ± 0.07 kcal/mol (P<<0.05) in the binding affinity (**Figure 4** and **Table 2**).

**Figure 5:**
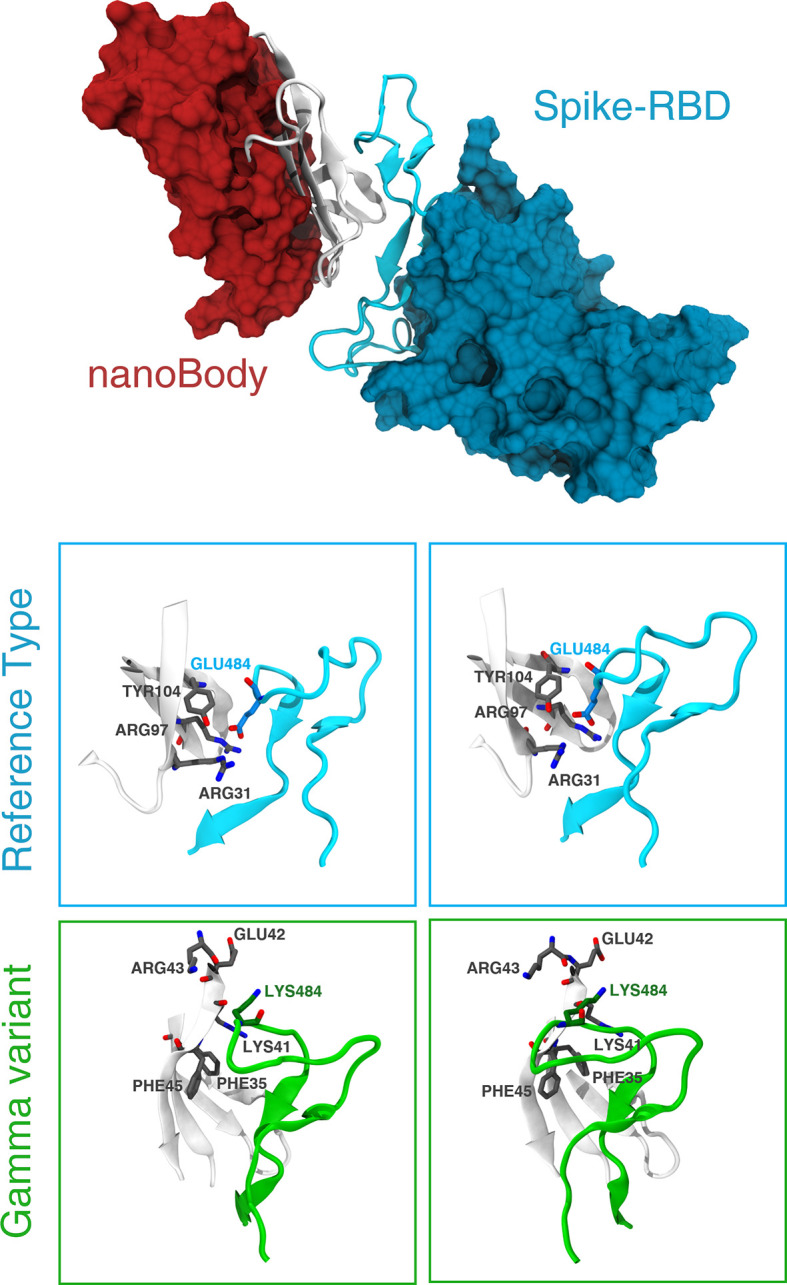
Details of the interaction between the nanobody and the gamma variant. The top panel shows the binding between the S-RBD region of the RT and the neutralizing nanobody Nb20. The interface of interaction is shown in cartoon representation (white for the Nb20 and cyan for S-RBD), while the rest of the protein is represented according to its surface (red for Nb20 and blue for S-RBD). The bottom panels show the differences between RT (cyan) and gamma variant (green) in stereographic representation. The positively charged Arg31 recognizes the negatively charged Glu484 in S-RBD in Nb20. This bond is clearly broken after the E484K mutation.

A detailed analysis of the nanobody-S-RBD complex trajectory shows that the change in binding energy is primarily due to mutation E484K. Residue GLU484, indeed, is located inside a positively charged pocket and stably interacts with the side chain of two arginines and a tyrosine (Arg97, Arg31, and Tyr104 – **Figure 5**). These interactions are clearly disrupted by the mutation E484K that inverts the residue charge, forcing it out of the pocket. On the other hand, there are no notable differences in the interaction of the two miniproteins between the RT and gamma variants.

### Experimental Validation of Computational Results on Single Point Variants

**Figure 6:**
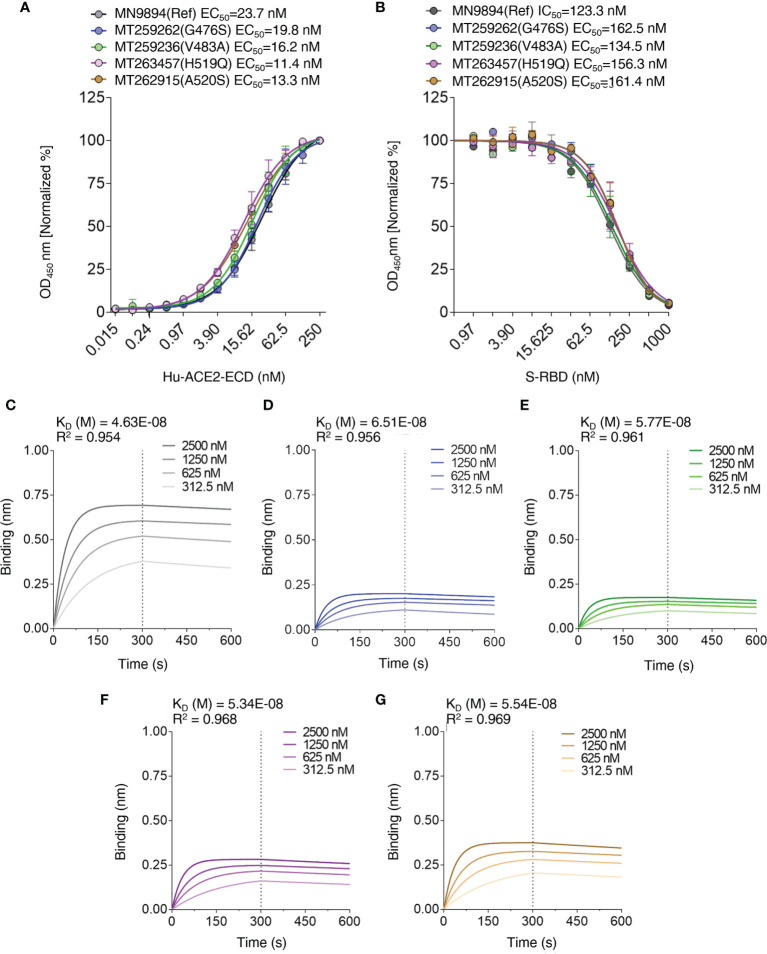
Interaction profiles of S-RBD reference/mutants and hACE2-ECD. **(A)** *EC_50_* values of S-RBD – hACE2-ECD interaction were determined by direct binding ELISA using titration curves with hACE2-ECD dilutions, and **(B)** *IC_50_* values of S-RBD – hACE2-ECD interaction were determined by competitive ELISA using titration curves with S-RBD dilutions and hACE2-ECD at a predetermined *EC_50_*. Biolayer interferometry was used to generate association and dissociation curves of S-RBD – hACE2-ECD interaction for S-RBD reference **(C)**, G476S **(D)**, V483A **(E)**, H519Q **(F)**, and A520S **(G)**; legends represent the nanomolar (nM) concentration of hACE2-ECD and *K_D_* values are depicted.

To validate the computational predictions, we performed an experimental comparative binding analysis using direct-binding and competitive ELISA experiments to determine *EC_50_/IC_50_* values for S-RBD – hACE2-ECD interaction. This analysis is limited to single-point variants. To determine *EC_50_* values of hACE-ECD binding to S-RBD reference/mutants, respective titration curves were generated using hACE-ECD dilutions on immobilized S-RBD protein (**Figure 6A**). Human ACE-ECD had a higher *EC_50_* against S-RBD reference (23.75 nM) in comparison to the mutants (11.48 – 19.86 nM); however, this difference was only 1.19 to 2.06-fold. To determine *IC_50_* of S-RBD – hACE2-ECD interaction, competitive binding reactions were set up by mixing hACE-ECD at a predetermined *EC_50_* with dilutions of respective S-RBD protein. Human ACE-ECD bound to S-RBD mutants with a slightly higher *IC_50_* (slightly lower apparent affinity) than S-RBD reference. We observed a statistically significant difference in the binding affinity of G476S (*p=0.002*) and A520S (*p=0.007*) S-RBD mutants compared to the reference (**Figure 6B**). However, consistent with the trend observed in *EC_50_* values, the fold difference in *IC_50_* for S-RBD reference/mutants was low (1.09 to 1.31). See **Table 3** for a summary of the results.

**Table 3:** ELISA binding profiles of S-RBD reference/mutants and hACE2-ECD.

	EC_50_ nM	EC_50_ Error ( ± SD)	IC_50_ nM	IC_50_ Error ( ± SD)
Ref	23.7	1.05	123.21	1.05
G476S	19.86	1.07	162.55	1.04
V483A	16.25	1.07	134.58	1.06
H519Q	11.48	1.07	156.31	1.06
A520S	13.39	1.07	161.43	1.04

Geometric mean EC_50_ (double replicates; n=3 independent experiments) and IC_50_ (double replicates; n=2 independent experiments) values of S-RBD reference/mutants and hACE2-ECD interaction are tabulated with standard error of the mean ( ± SD).

To further validate the binding trend obtained by ELISA *IC_50_* values, biolayer interferometry (BLI) kinetics experiments were performed by loading HIS1K sensors with S-RBD reference/mutants at a concentration of 1000 nM, and hACE-ECD (two-fold dilutions; 2500 – 312.5 nM) was used as analyte. Consistent with ELISA *IC_50_* trend, the calculated equilibrium dissociation constants (*K_D_)* range for S-RBD – hACE-ECD interaction was narrow (Ref – 46.3 ± 1.28 nM, G476S – 65.1 ± 1.17 nM, V483A 57.7 ± 0.96 nM, H519Q 53.4 ± 0.94 nM, and A520S 55.4 ± 1.05 nM) (**Figures 6C–G**). A statistically significant difference was observed in the *K_D_* values of G476S and V483A, compared to reference S-RBD. Furthermore, like *EC_50_* and *IC_50_*, the *K_D_* values differed on an average from 1.11 to 1.39-fold from the S-RBD reference. Further, the on (*K_ON_ =* 9.86 x 10^3^ to 1.11 x 10^4^ 1/Ms) and off-rates (*K_DIS_ =* 4.57 x 10^-4^ to 6.68 x 10^-4^ 1/s) were largely similar for all S-RBD samples. The binding behavior of selected S-RBD mutants seems less likely to modulate S-RBD – hACE-ECD interaction. The experiments were performed in three replicas and results are summarized in **Table 4**.

**Table 4:** Kinetics profiles of S-RBD reference/mutants and hACE2-ECD.

	K_D_ (M)	K_D_ Error	K_ON_ (1/Ms)	K_ON_ Error	K_DIS_ (1/s)	K_DIS_ Error	Full R**^2^**
**Rep-1**							
Ref	4.63E-08	1.34E-09	9.86E+03	6.19E+01	4.57E-04	1.29E-05	0.9543
G476S	6.51E-08	1.32E-09	1.03E+04	6.39E+01	6.68E-04	1.29E-05	0.9565
V483A	5.77E-08	1.11E-09	1.11E+04	6.40E+01	6.39E-04	1.18E-05	0.9618
H519Q	5.34E-08	1.05E-09	1.07E+04	5.63E+01	5.71E-04	1.08E-05	0.9688
A520S	5.54E-08	1.14E-09	9.91E+03	5.24E+01	5.49E-04	1.09E-05	0.9694
**Rep-2**							
Ref	4.62E-08	1.21E-09	1.10E+04	7.04E+01	5.08E-04	1.29E-05	0.95
G476S	6.32E-08	1.03E-09	1.29E+04	7.96E+01	8.17E-04	1.23E-05	0.9544
V483A	4.78E-08	8.25E-10	1.39E+04	7.82E+01	6.65E-04	1.09E-05	0.9606
H519Q	4.96E-08	8.50E-10	1.30E+04	6.94E+01	6.45E-04	1.05E-05	0.965
A520S	5.42E-08	9.59E-10	1.16E+04	6.15E+01	6.30E-04	1.07E-05	0.9672
**Rep-3**							
Ref	4.16E-08	1.12E-09	1.21E+04	8.07E+01	5.03E-04	1.32E-05	0.9428
G476S	5.70E-08	9.72E-10	1.42E+04	9.33E+01	8.11E-04	1.28E-05	0.9459
V483A	4.50E-08	7.97E-10	1.53E+04	9.34E+01	6.91E-04	1.15E-05	0.9522
H519Q	4.58E-08	8.27E-10	1.44E+04	8.49E+01	6.60E-04	1.13E-05	0.9554
A520S	4.96E-08	9.09E-10	1.29E+04	7.26E+01	6.38E-04	1.11E-05	0.9607

Experimental K_D_ values in molar (M) concentration, association rate constant [K_ON_ (1/Ms)], and dissociation rate constant [K_DIS_ (1/s)] with error generated while fitting the binding curves from three replicates for S-RBD reference/mutants and hACE2-ECD interaction are tabulated.

Comparison with computational estimates can be done using the formula $\Delta \Delta G_{Exp}=RT\Delta \text{ln}\left(K_{D}\right)$ (see method Section) where *K_D_* is the average obtained from the 3 replicas. The correlation between the two set of data is R=0.996 (**Supplementary Figure 2**) showing that our method is able to achieve a qualitative correct prediction of the effect of the mutations on the binding affinity.

## Discussion

In this paper, we performed an *in silico* screening of a selected number of SARS-COV2 variants and calculated the binding affinity between S-RBD and the hACE2 receptor. Results of the simulations, in agreement with experimental observations, do not show remarkable differences in the expected *K_D_* for any of the variants. In some cases, variants show even a worsened affinity.

However, variations in *K_D_* not necessarily translate into a higher infectivity of the virus. Many different effects might be in play, and even mutations far from the binding site can improve the virus’s fitness. The D614G mutation constitutes a clear example. This variant introduced a mutation far away from the ACE2 interaction domain and displaced the original variant isolated in Wuhan worldwide in a couple of months. Similar effects have been recently reported for other mutations away from the recognition domain (55).

Notwithstanding, the *K_D_* remains a key factor to be analyzed. This is the reason why we think our contribution can be helpful to other researchers working in the design and identification of miniproteins and nanobodies against SARS-CoV-2 (52, 54, 56, 57). Having a preliminary screening of the effect of mutations on the binding affinity can help save time and other resources, especially during emergency situations, like the one we are experiencing in the current pandemic.

The great diffusion of newer virus variants (58) suggests an evolutionary advantage due to the mutations, even though their affinity is not significantly changed. Several recent studies indicate that these mutations could lead to immune escape (32–35).

To have an idea on how immune escape could happen at molecular level, we analyzed MD trajectories and computed the binding free energy of the gamma variant bound to a highly specific nanobody. We found that the affinity is indeed reduced by 1.2 kcal/mol and that this change is due mainly to the E484K mutation. This mutation can be found in several emerging SARS-COV-2 variants, and was shown to affect the binding of antibodies significantly. In other words, the virus can trade its ability to tightly bind to the hACE2 receptor in exchange for becoming more elusive to specific antibodies.

While this mechanism cannot be generalized for the whole antibody population, we can see that position 484 is a good mutation spot for the virus, from an evolutionary point of view, since this residue only interacts with neutralizing antibodies and not with the hACE2 receptor. Indeed, other mutations have also been found in this position, such as the E484Q in the kappa variant. Spreading of similar variants could escape the antibody recognition and could require a periodical update of vaccines and monoclonal antibodies used in clinical applications to avoid a potential loss of efficacy (34).

On the other hand, synthetic miniproteins that have been designed to mimic the structure of the hACE2 receptor, the natural binder for the S protein, are less affected by the mutation, and they still can work as bait.

It is worth to notice that the absolute values of the binding energies calculated may depend on some of the computational details chosen (namely, force field, water models, specific methods to calculate binding energies, etc.). However, our method is able to achieve a qualitatively correct prediction of the effect of mutations at the protein-protein interface. Indeed, we obtained a high correlation with analogous values calculated using the MM-PBSA and with experimental results (**Supplementary Figure 1**).

In summary, our work demonstrated that molecular simulations can be used to rapidly screen the effect of SARS-COVID-2 mutations, in particular concerning their ability to bind the hACE2 receptor or neutralizing molecules. This kind of analysis could be of primary importance as a preliminary screening and to produce working hypotheses that can help to prioritize the experimental study on the virus mutations.

## Data Availability Statement

The raw data supporting the conclusions of this article will be made available by the authors upon request, without undue reservation.

## Author Contributions

DB built structural models and performed molecular dynamics simulations. QJ produced S-RBD and hACE2-ECD proteins and performed binding experiments by ELISA and BLI. DB, QJ, AS, GY, SP, and FZ analyzed data. DB, AS, and FZ wrote the first draft of the manuscript. FZ and SP wrote the final version of the manuscript. AS and GY supervised wet-lab experiments. FZ and SP supervised computational studies. All authors contributed to editing and approved the final draft. 

## Funding

This work was supported by the National Science Foundation of China (Grant No. 31770776 to FZ) and by FOCEM (MERCOSUR Structural Convergence Fund, COF 03/11 to SP).

## Conflict of Interest

The authors declare that the research was conducted in the absence of any commercial or financial relationships that could be construed as a potential conflict of interest.

## Publisher’s Note

All claims expressed in this article are solely those of the authors and do not necessarily represent those of their affiliated organizations, or those of the publisher, the editors and the reviewers. Any product that may be evaluated in this article, or claim that may be made by its manufacturer, is not guaranteed or endorsed by the publisher.

## Supplementary Material

Supplementary Figure 1 Correlation between binding free energy computed by MM-PBSA and the PRODIGY webserver.

Supplementary Figure 2 Comparison of binding affinity computed using the PRODIGY webserver and available experimental data. Binding affinities ratios are obtained from the dissociation constant using the formula: *RT*Δ ln(*K_D_*) = ΔΔ*G_Exp_*. (top) Table containing *K_D_*, and ΔΔG, ΔΔG_Exp_ and standard error of the mean computed using the RT as reference. (bottom) Correlation between binding affinity computed using PRODIGY and ΔΔG_Exp_.

Supplementary Figure 3 Root mean square deviations (RMSD) of the complex S-RBD hACE2 receptor simulation. We report here only one (randomly chosen) of the five trajectory replicas produced for data analysis. All other simulations produce similar results.

Supplementary Figure 4 Details of the interaction of the RT S-RBD and hACE2 receptor. For clarity, the figure is split into two different regions, which are spatially separated **(A, B)**. Lys417 interacts only with Asp30. Tyr449 and Gln498 share an interaction with Asn38 and Gln42 side chains. Leu455 and Phe456 are inside a pocket of charged amino acids and interact with the main chain of Asp30, Lys31, and Thr27. Phe486 is inside a hydrophobic pocket formed by Phe28, Leu79, and Phe83. Asn487 interacts with the polar side chain of Gln24. Gln493 is within a charged pocket and interacts with the side chains of Lys31, Glu35, and His34. Thr500 shares an interaction with the polar side chain of Tyr41 and the charged side chains of Asp355 and Arg357. Asn501 is inside a charged pocket but interacts to some extent with Tyr41 and Lys353. Tyr505 establishes an interaction with Glu35 and Arg393.

Supplementary Figure 5 Maps of the contact between different variants of S-RBD and hACE2 receptor. The x-axis of the matrix represents S-RBD residues, while the y axis represents hACE2 receptor residues. The color represents the probability of interaction along the whole set of trajectories and goes from white (0%) to blue (100%).
