# Supplementary figures and images for: Rapid Assessment of Binding Affinity of SARS-COV-2 Spike Protein to the Human Angiotensin-Converting Enzyme 2 Receptor and to Neutralizing Biomolecules Based on Computer Simulations
Source: Front Immunol. 2021 Nov 11;12:730099. doi: 10.3389/fimmu.2021.730099 (PMC8632240; doi:10.3389/fimmu.2021.730099)

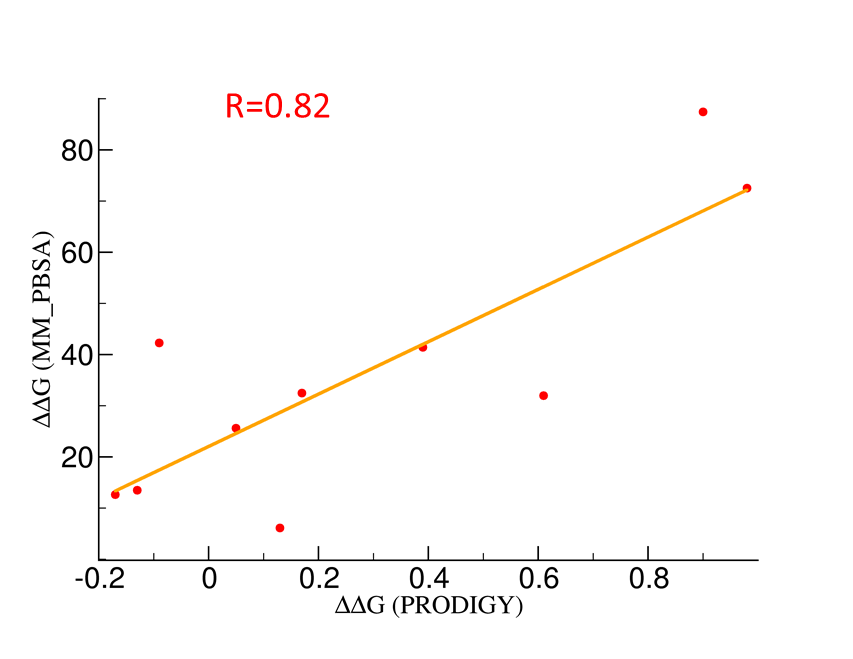

Supplement: Supplementary Figure 1 — Correlation between binding free energy computed by MM-PBSA and the PRODIGY webserver. [file Image_1.tif]

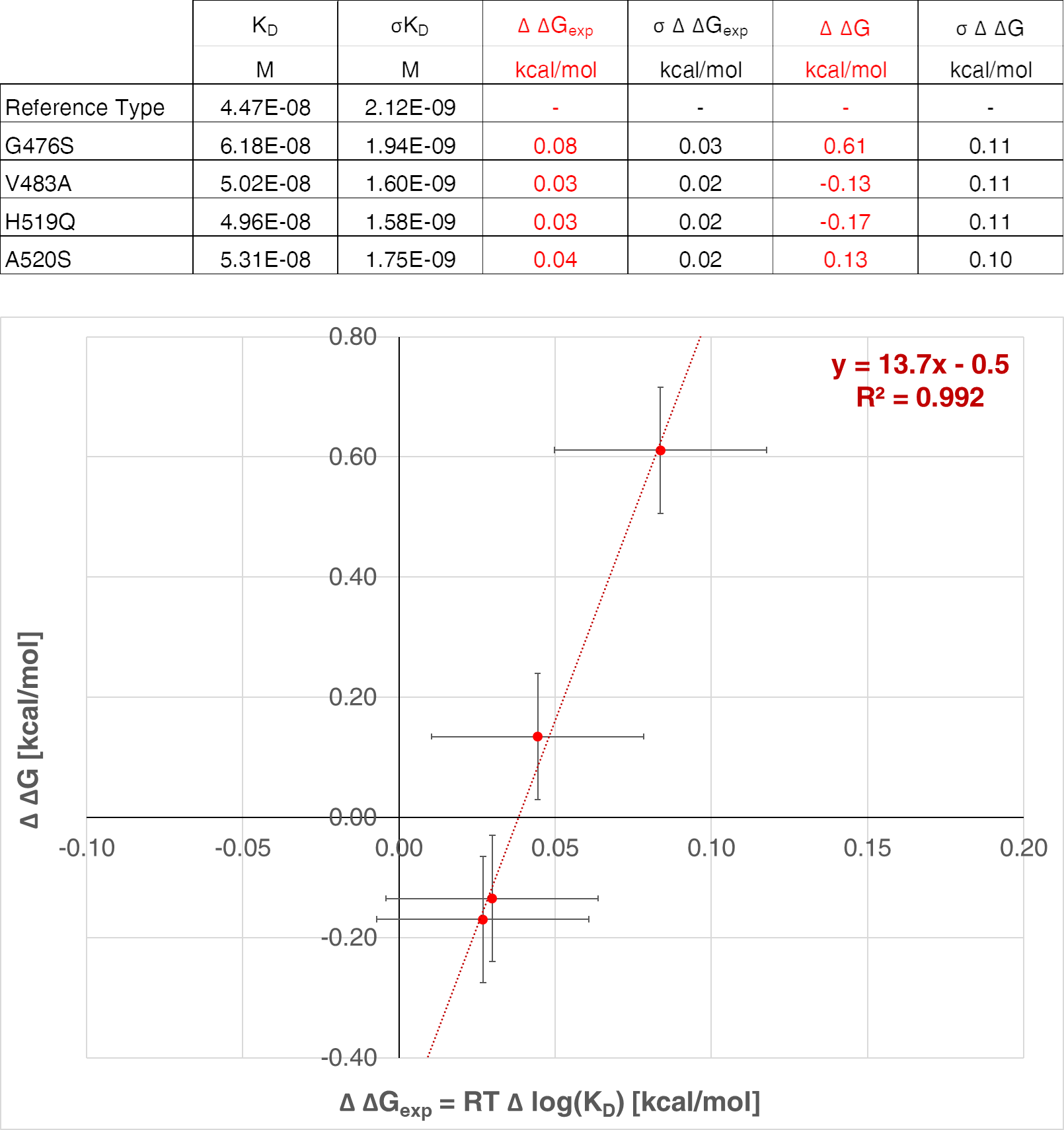

Supplement: Supplementary Figure 2 — Comparison of binding affinity computed using the PRODIGY webserver and available experimental data. Binding affinities ratios are obtained from the dissociation constant using the formula: RTΔ ln(KD ) = ΔΔGExp . (top) Table containing KD , and ΔΔG, ΔΔGExp and standard error of the mean computed using the RT as reference. (bottom) Correlation between binding affinity computed using PRODIGY and ΔΔGExp. [file Image_2.tif]

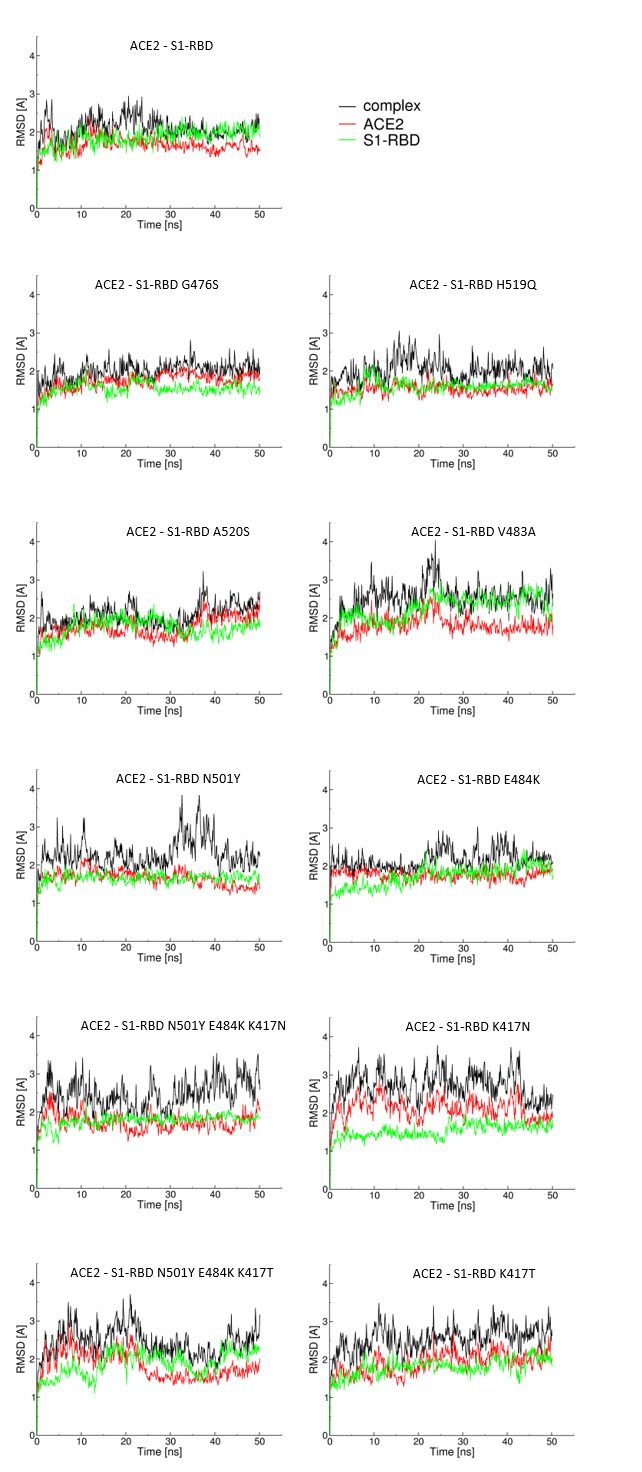

Supplement: Supplementary Figure 3 — Root mean square deviations (RMSD) of the complex S-RBD hACE2 receptor simulation. We report here only one (randomly chosen) of the five trajectory replicas produced for data analysis. All other simulations produce similar results. [file Image_3.tif]

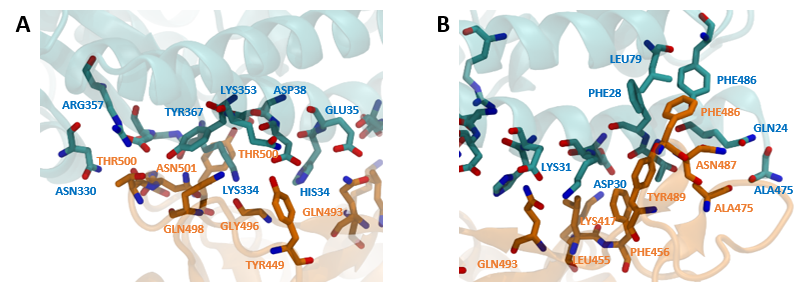

Supplement: Supplementary Figure 4 — Details of the interaction of the RT S-RBD and hACE2 receptor. For clarity, the figure is split into two different regions, which are spatially separated (A, B). Lys417 interacts only with Asp30. Tyr449 and Gln498 share an interaction with Asn38 and Gln42 side chains. Leu455 and Phe456 are inside a pocket of charged amino acids and interact with the main chain of Asp30, Lys31, and Thr27. Phe486 is inside a hydrophobic pocket formed by Phe28, Leu79, and Phe83. Asn487 interacts with the polar side chain of Gln24. Gln493 is within a charged pocket and interacts with the side chains of Lys31, Glu35, and His34. Thr500 shares an interaction with the polar side chain of Tyr41 and the charged side chains of Asp355 and Arg357. Asn501 is inside a charged pocket but interacts to some extent with Tyr41 and Lys353. Tyr505 establishes an interaction with Glu35 and Arg393. [file Image_4.tif]

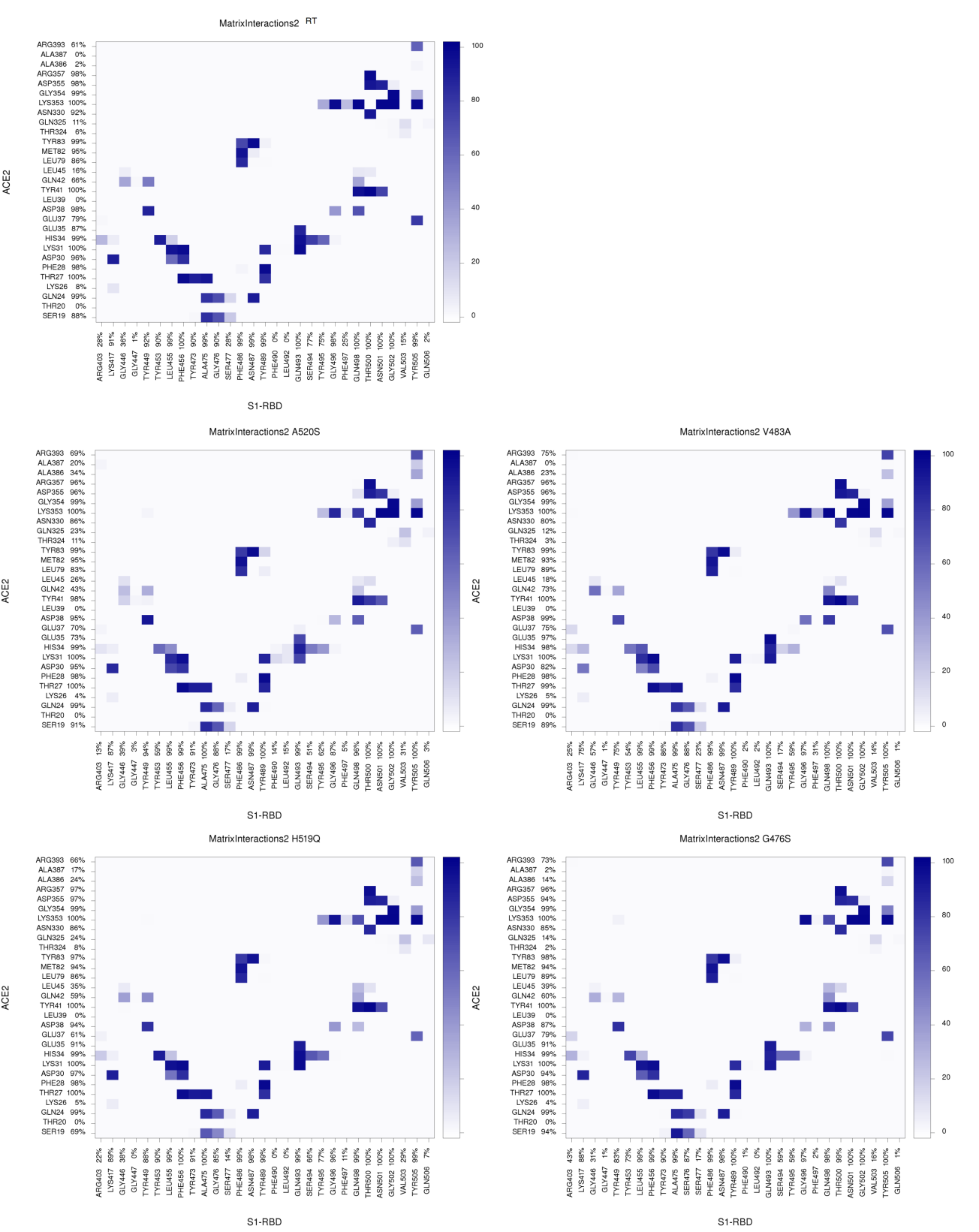

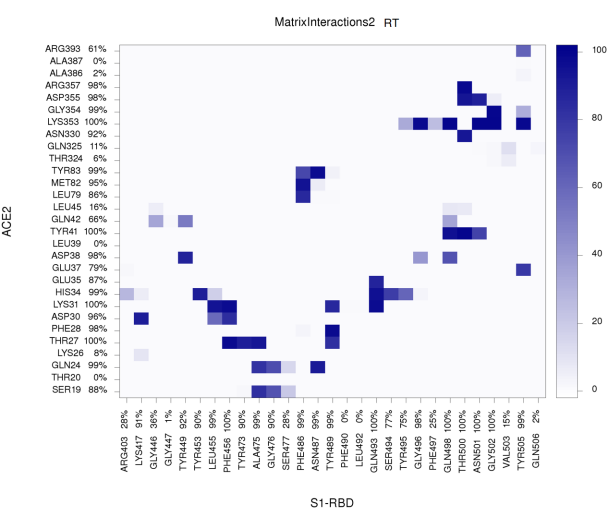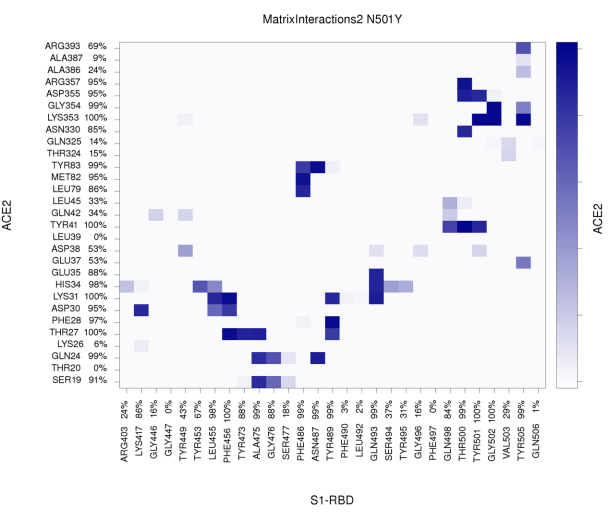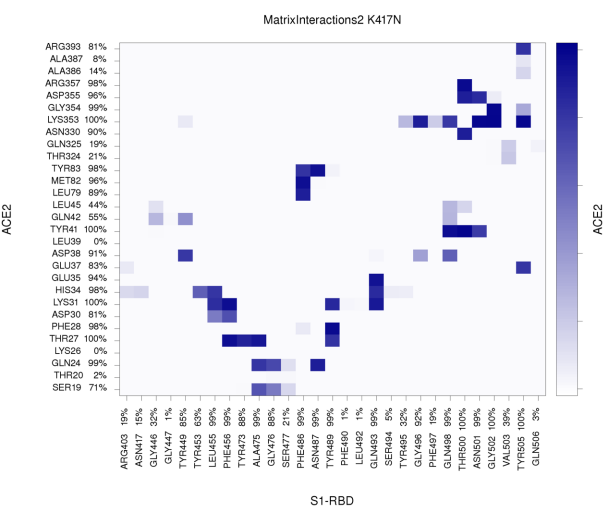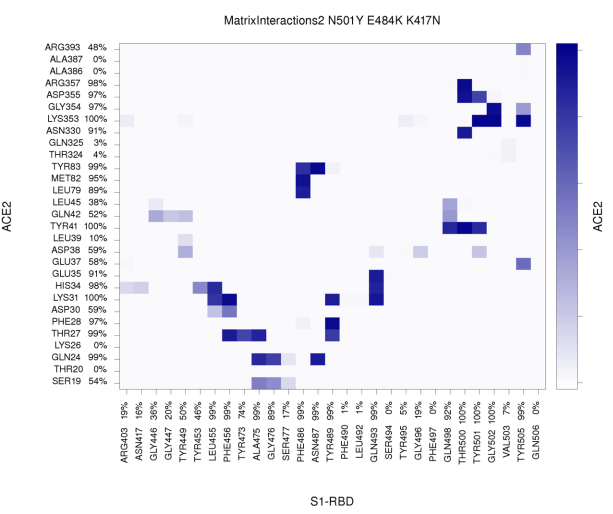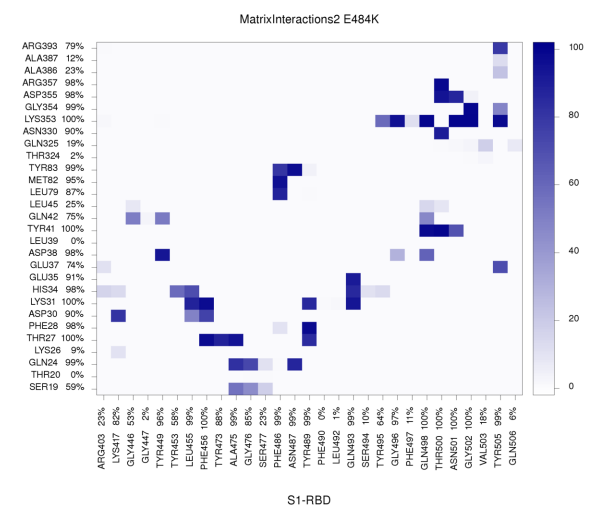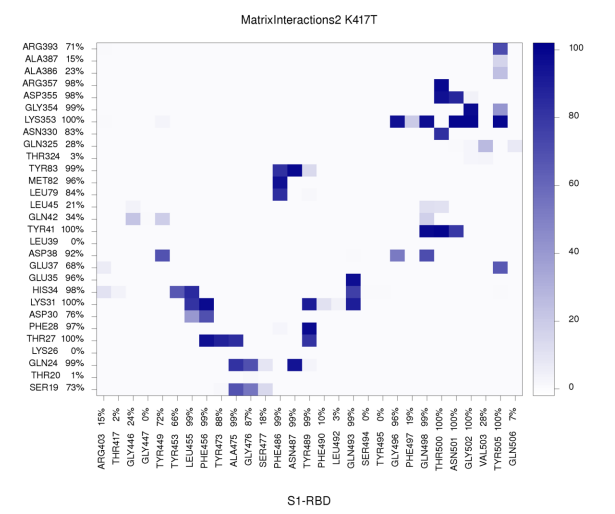

Supplement: Supplementary Figure 5 — Maps of the contact between different variants of S-RBD and hACE2 receptor. The x-axis of the matrix represents S-RBD residues, while the y axis represents hACE2 receptor residues. The color represents the probability of interaction along the whole set of trajectories and goes from white (0%) to blue (100%). [file Image_5.pdf]
